# “Touch&Play” Wood Typification by the MasSpec Pen

**DOI:** 10.1002/jms.5133

**Published:** 2025-05-29

**Authors:** Adriano Reis, Caroline Pais Carvalho, Iasmim Lopes de Lima, Felipe R. P. Mansoldo, Alane Beatriz Vermelho, Rosineide Costa Simas, Livia S. Eberlin, Marcos N. Eberlin

**Affiliations:** ^1^ School of Engineering Mackenzie Presbyterian University São Paulo São Paulo Brazil; ^2^ MackGraphe—Mackenzie Institute for Research in Graphene and Nanotechnologies Mackenzie Presbyterian Institute São Paulo São Paulo Brazil; ^3^ BIOINOVAR—Biotechnology Laboratories: Biocatalysis, Bioproducts and Bioenergy Institute of Microbiology Paulo de Góes, Federal University of Rio de Janeiro (UFRJ) Rio de Janeiro Rio de Janeiro Brazil; ^4^ Department of Surgery Baylor College of Medicine Houston TX USA

**Keywords:** ambient massspectrometry, andiroba, Brazilian mahogany, cedar, mass spectrometry, MasSpec Pen, wood

## Abstract

We have investigated the ability of a MasSpec Pen (MSPen) “three‐in‐one” (extraction, transfer, and ionization) device coupled to a mass spectrometer to provide instantaneous chemical profiles that could promptly characterize wood samples from the mahogany (*Meliaceae)* family. For that, we selected a set of five representative wood species, that is, Brazilian mahogany ( *Swietenia macrophylla* , also known as Honduran mahogany), two African mahoganies (*Khaya ivorensis and Khaya senegalensis*), and two “nongenuine” (“fake”) mahogany woods: cedar ( *Cedrela odorata* ) and andiroba ( *Carapa guianensis* ). By simply touching the superficially polished wood surface and after 3 s of automatic extraction, profiles of highly characteristic markers that effectively discriminated all five mahoganies were detected. The superficial surface of a wood Brazilian mahogany sample as compared with internal wood accessed via deep sanding showed minor profile changes mainly by shifts in the relative abundances of the wood markers, indicating that aging only marginally changes MSPen wood signatures. The direct “touch&play” analysis offered by MSPen was therefore found to provide nondestructive, fast, sample‐preparation‐free, and reliable typification of woods. This “spatially free” device also allows broad screening because multiple points on the whole surface of any small or large‐size intricate wood sample can be rapidly analyzed, demonstrating its high potential for forensic investigations, particularly for endangered species such as the Brazilian mahogany.

## 1 Introduction

Woods are renewable, abundant, and essential for many industrial segments [1]. In Brazil, the intense exploration of this highly useful and exquisitely designed natural material has placed some valuable woods such as Pau‐Brasil ( *Paubrasilia echinata* ), rosewood ( *Dalbergia nigra* ), pine (*Araucaria augustifolia*), and Brazilian mahogany ( *Swietenia macrophylla* , also known as Honduran mahogany) on the list of endangered species [2, 3].

**FIGURE 1:**
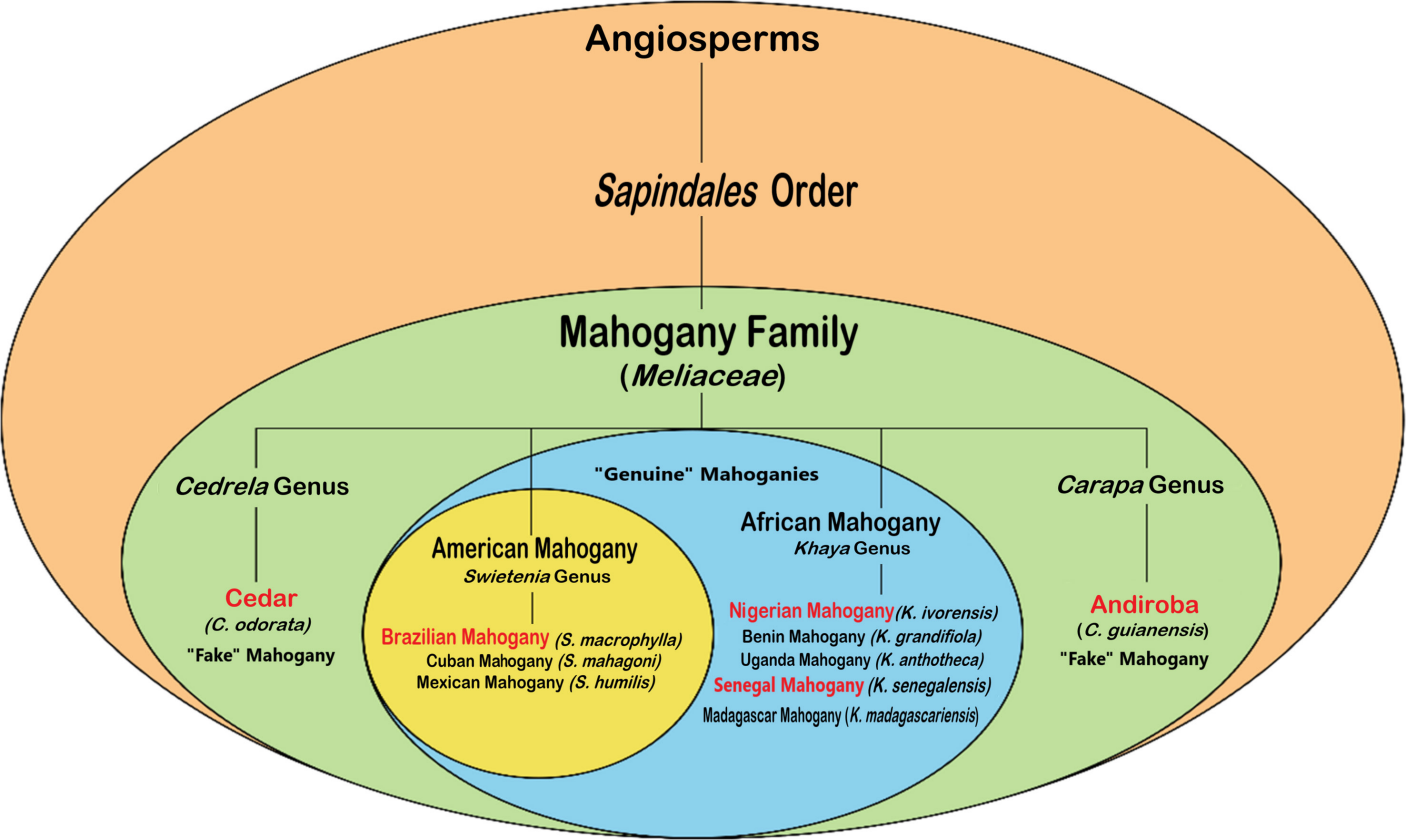
Botanical classification of wood species from the *mahogany* (*Meliaceae)* family according to genera and species, adapted from the Wood Database [5]. Note that the species investigated in this study are highlighted in red, whereas cedar and andiroba do belong to the mahogany family but are classified herein as “fake” mahogany because only woods from the *Swietenia* genus are considered to be “genuine” mahoganies. Note also that African and Philippine mahoganies (from the *Shorea* and *Parashorea* genus), from their close genus proximity, are also often classified as “genuine” mahoganies, so we have included the African mahoganies within the “genuine” group.

Brazilian mahogany is easily recognized by its characteristic reddish hue and long‐lasting and highly appreciated attributes, which have made it a preferred wood for opulent furniture, flooring, musical instruments, and ornamental artifacts. Due to its endangered status, logging, trading, and export [4] of Brazilian mahogany is currently illegal, but unfortunately other “nongenuine” (“fake”) tropical mahoganies, such as andiroba ( *Carapa guianensis* ) and cedar ( *Cedrela odorata* ), from closely related genera (Figure 1), show remarkable morphological similarities to the “genuine” Brazilian mahogany. Therefore, to escape persecution, this endangered species is frequently commercialized as if it was cedar or andiroba [4, 6, 7]. This misidentification undermines conservation efforts and threatens the endangered Brazilian mahogany.

In Brazil, mainly two African mahoganies, that is Nigerian mahogany (*Khaya ivorensis*) and Senegal mahogany ( *Khaya senegalensis* ), have been cultivated as substitutes for Brazilian Mahogany, aiming to reduce its illegal extraction [8]. These most closely related mahoganies (Figure 1), also often considered “genuine” mahoganies, provide hardwoods with similar physical and mechanical properties as those of Brazilian mahogany [9].

Tools capable of quickly, consistently and broadly typifying these mahoganies would therefore prove highly valuable in mitigating illegal logging and trading of woods, particularly concerning the endangered Brazilian mahogany, as well as combating counterfeiting while facilitating the establishment of certified wood standards [10, 11].

Macroscopic and microscopic inspection reveals texture, odor, color, density, cellular dimensions, type of ornamentation of cell walls, and cellular composition of parenchymal rays [12, 13] of woods. But differences within species or even genera are often subtle, and therefore, taxonomical classification of wood heavily depends on specialized professionals, sometimes leading to false positives or negatives [7, 14]. Near infrared (NIR) spectroscopy is widely used for wood typification, but this technique displays some crucial drawbacks, such as time‐consuming calibration models [15, 16], sensitivity to physical variability [17], similar spectra for most closely‐related woods, challenges in field conditions due to moisture and portability issues [18], and interference from extractive compounds, which call for coupling with other techniques such as thin‐layer chromatography (TLC) [19, 20].

Previous works have indicated that molecular fingerprinting by mass spectrometry (MS) could function as the gold‐standard technique for wood typification, because MS characterization is based on unmistakable pools of biomolecular makers. MS has demonstrated therefore superior performance, and unique molecular profiles for many species of woods have been reported [21, 22]. For this application, ambient MS stands out, enabling even faster and direct “touch&play” wood analysis with minimal or no sample processing [23, 24]. Direct analysis in real time‐MS (DART‐MS), for example, which is also based on pool of unique molecular markers, has proven effective in typifying wood samples [23, 25, 26, 27], and a curated reference database (ForeST Database©) of about 2000 woods has been developed [28].

MS techniques for sampling and ionization such as Venturi easy ambient sonic spray ionization (V‐EASI) in either its solid (V_S_‐EASI) or liquid (V_L_‐EASI) modes have also provided distinct wood profiles of molecular markers, offering secure MS typification, as shown for Brazilian mahogany and Nigerian mahogany cultivated in Brazil [24]. But V_L_‐EASI‐MS still requires sampling (removal of a small wood shard) and prior solvent extraction, whereas V_S_‐EASI‐MS, similarly to DART‐MS, has spatial constraints particularly for large samples with multiple parts because target spots on the wood surface must be positioned very close to the ion source.

The MasSpec Pen (MSPen) has been introduced as a highly efficient “touch&play” direct, nondestructive, “three‐in‐one” (solvent extraction plus transfer and ionization) device designed for “sample‐preparation free” instantaneous MS analysis [29]. MSPen is also considerably “spatially free", allowing widespread sampling along large and multifaceted surfaces, because it can be moved freely around the sample within several meters from the ion source, enabling multiple spots on the sample surface to be exhaustively inspected within minutes [30]. This powerful device has therefore been used with success, particularly in medical MS, for in vivo and ex vivo tissue analysis, rapid COVID‐19 screening, noninvasive detection of chemical defenses in poison frogs, and identification of clinically relevant bacteria [31, 32, 33, 34]. In the forensic field, MSPen has been shown to enable nearly instantaneous and often semiquantitative chemical analysis of agrochemicals, explosives, and drugs, and to provide rapid analysis and authentication of meat [35, 36].

Herein, we describe an exploratory proof‐of‐principle study using a challenging set of five (“genuine” and “fake”) woods from the same mahogany family (Figure 1) aimed to evaluate the ability of MSPen to typify woods.

## 2 Materials and Methods

### 2.1 Materials

Methanol HPLC grade (LiChrosolv) and acetonitrile HPLC grade (Sigma‐Aldrich) were purchased from Merck (Darmstadt, Germany).

Certified samples of wood from Andiroba, Cedar, and Brazilian mahogany were provided by the Laboratory of Forest Products (Brasília, Brazil). Nigerian and Senegal mahoganies were provided by the Xilotheque Dr. Calvino Mainieri of the Institute for Technological Research (BCTw‐IPT, São Paulo, Brazil). Biological replicates consisting of five samples from different trees were analyzed for each wood species, except for the Senegal mahogany, for which only a single sample was available.

### 2.2 MasSpec pen

A MSPen prototype system [29] coupled to an LTQ XL Ion Trap mass spectrometer (Thermo Scientific, San Jose, CA, USA) was used, with solvent delivery provided by a Fusion 101 syringe pump (Chemyx Inc., Stafford, USA). Main conditions were as follows: 30 μL solvent droplet volume, 1 m tubing length, 2200 ms infusion time, 800 μL/min infusion rate, and 15 s transport time. We tested different extraction times from 3 to 5 s, using different solvents or solvent mixtures (MeOH:CH_3_CN) for extraction. A clean system (tubing, pen tip, and capillary transfer tube) was used for each sample, and a blank background spectrum was acquired before analysis. For each wood sample, 3 to 4 different surface spots were analyzed per sample, with each spot treated as a technical replicate.

The mass spectrometer was operated with the following main conditions: capillary temperature of 350°C, tube lens offset of 100 V, and capillary voltage of 35 V. Mass spectra were acquired in the profile, full scan, and positive ion modes, within a mass range of *m/z* 200 to 2000.

### 2.3 Data Analysis

Raw files were converted to mzML by msConvert (ProteoWizard 3.0, Palo Alto, Canada) with 32‐bit encoding precision. Data processing was performed in the R environment [37] by using our recently developed rIDIMS package [38] to select high‐quality scans, perform data binning, and filter ions. Technical replicates were averaged, ions were filtered at 5% abundance, and data were binned at *m/z* = 1. Ions were removed if their abundances were less than three times that of the blank, and only ions present in at least three out of five biological samples from each wood species were retained in the final data matrix.

For multivariate analysis, the dataset was initially subjected to a feature selection step by using the BORUTA algorithm, implemented through the Boruta R package [39]. The resized dataset, containing only the important selected ions (variables), was formatted for compatibility with the MetaboAnalyst platform, with samples in rows and *m/z* values (features) in columns. The data were then processed using the R package MetaboAnalystR [40] and normalized using row‐wise normalization (SumNorm) and data scaling (AutoNorm). Principal component analysis (PCA), PCA biplot, and hierarchical clustering heatmap analysis were performed subsequently.

## 3 Results and Discussion

### 3.1 MSPen Data

First, we optimized the extraction time and solvent composition for best molecular analysis of the woods. All MSPen data were collected in the positive ion mode because prior studies have demonstrated its effectiveness to detect unique wood markers [22, 24]. The optimal overall extraction time was 3 s, and a MeOH:CH_3_CN (90:10, v/v) solvent exhibited the most favorable overall balance between sample and background ions.

**FIGURE 2:**
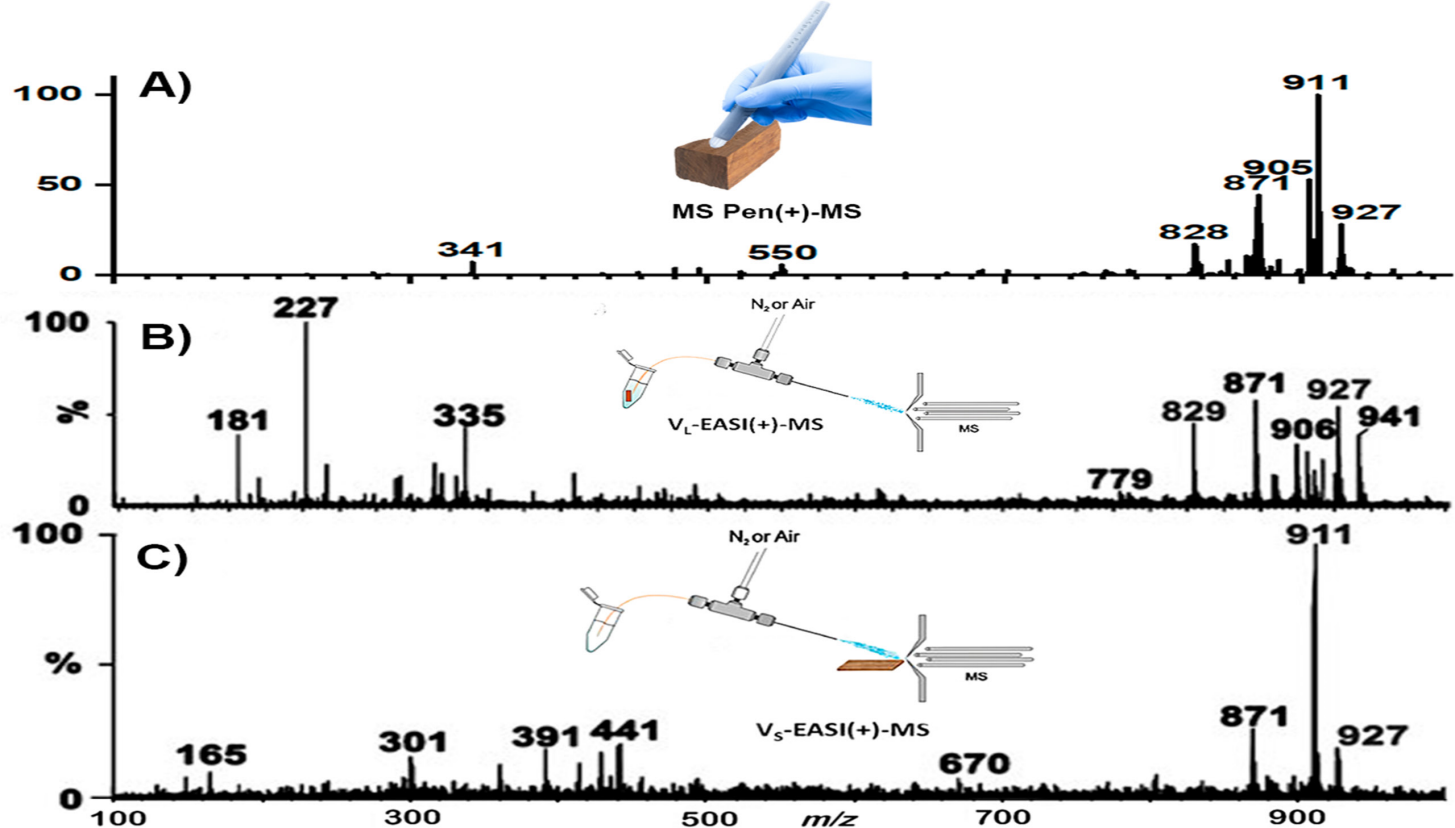
Representative (A) MSPen(+); (B) V_L_‐EASI(+)‐MS; and (C) V_s_‐EASI(+)‐MS in the positive ion mode (+) for Brazilian mahogany obtained using pure MeOH as the solvent. Note that (B) and (C) have been adapted from Cabral et al. [24]. For comparison, schematics of these techniques are also shown as inserts.

First, we noted that the MSPen, although so rapidly and undisturbedly touching the wood surface, yielded highly distinct and reproducible MS profiles for Brazilian mahogany (Figure 2A), similarly to those previously reported for V_L_‐EASI‐MS and V_s_‐EASI‐MS [24]. Likely due to ultrafast extraction (3 s), the MSPen profile (Figure 2A) most closely resembles the V_S_‐EASI‐MS profile (Figure 2C), whereas the V_L_‐EASI‐MS profile (Figure 2B) reflects its more exhaustive solvent extraction nature with a more diverse set of makers. In fact, the MSPen profile of Figure 2A can be viewed half‐way from Figure 2B and 2C. Note that in Figure 2A exclusively, for comparison, MSPen acquisition parameters were adjusted to most closely match those reported for V‐EASI‐MS [24], including the use of pure MeOH as the extraction solvent.

**FIGURE 3:**
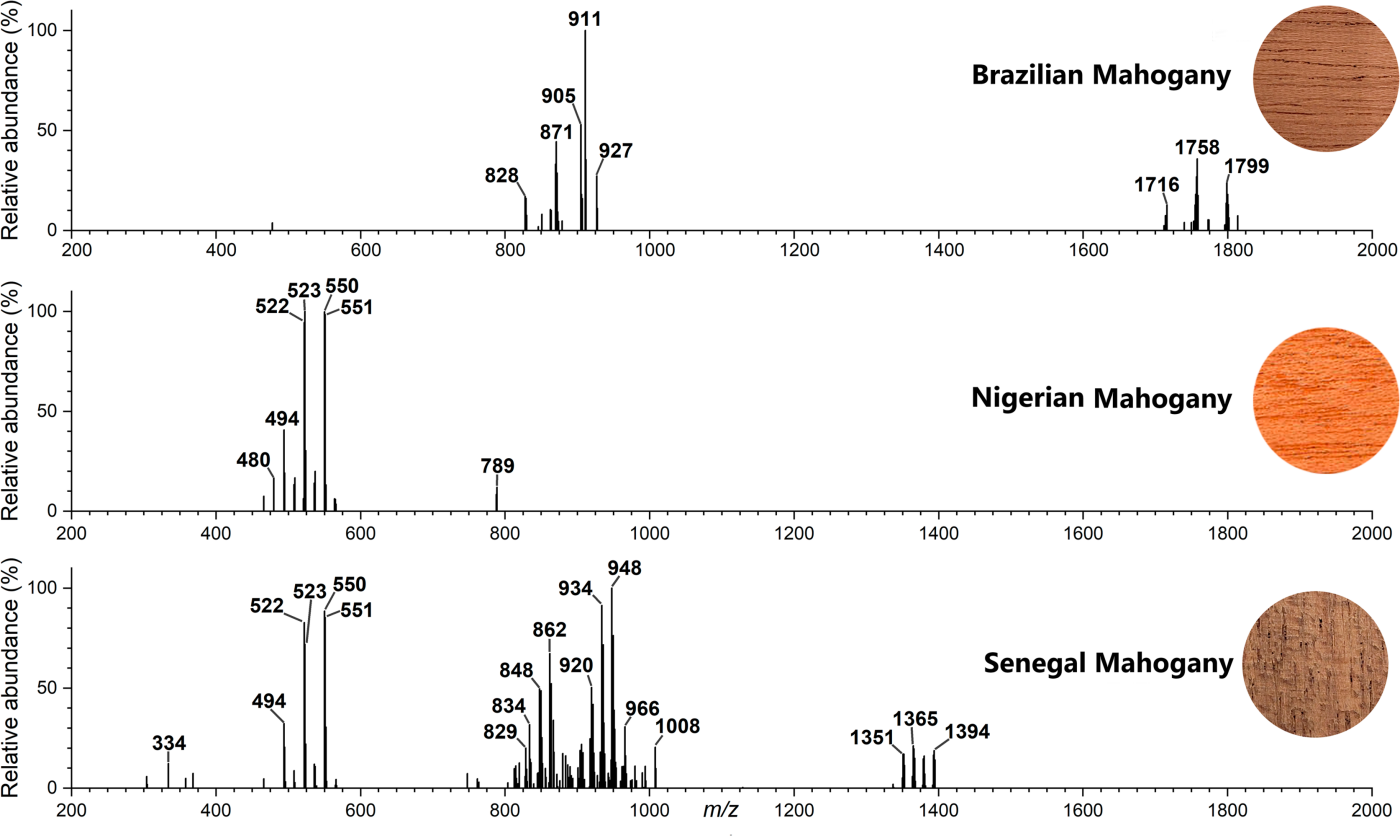
Representative MSPen(+) for (A) Brazilian mahogany, (B) Nigerian mahogany, and Senegal mahogany.

MSPen was then tested under its optimized conditions (MeOH:CH_3_CN, 90:10 vv, 3 s of extraction) for the three most closely‐related “genuine” Mahogany samples: American Brazilian and the two African Nigerian and Senegal mahoganies (Figure 3). Indeed, despite their very close taxonomic proximity, very distinct profiles were obtained for each sample, most particularly when the American and African genera are compared. Note for the American Brazilian mahogany (Figure 3A) a very unique ion package within *m/z* 828–927 as well a second and also very unique ion package at a beneficial (less interference from contaminants and other chemical noise) high mass region of *m/z* 1716–1799. Note that this second ion package for the Brazilian mahogany was unnoticed before. The two African mahoganies (Figure 3B,C) share a characterized package of ions within *m/z* 480–551, but the Nigerian mahogany displays a “solitary ion” of *m/z* 789, whereas the Senegal mahogany displays two additional unique ion packages within *m/z* 829–1008 and *m/z* 1351–1394.

**FIGURE 4:**
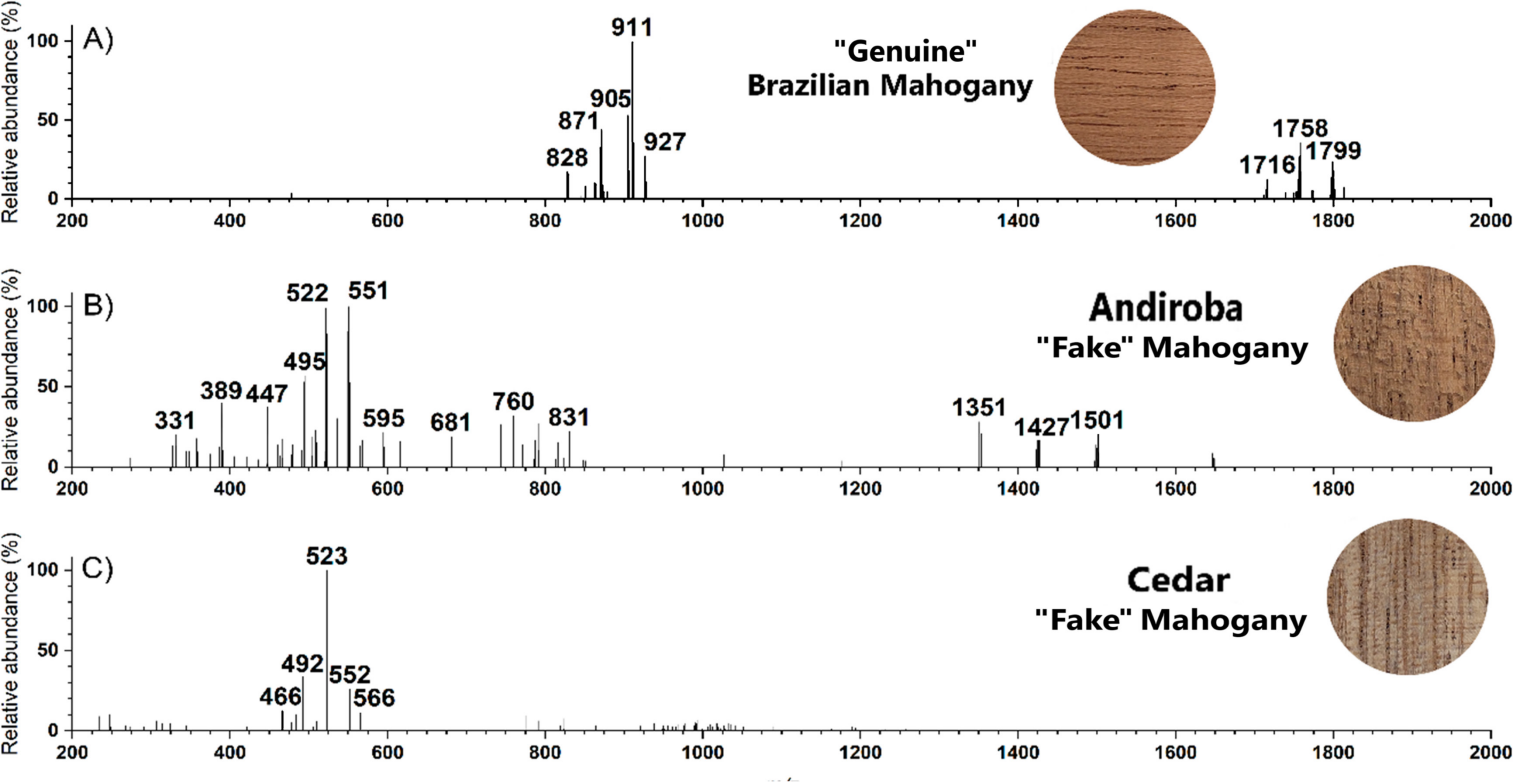
Representative MSPen(+)spectra of (A) Brazilian mahogany, (B) andiroba, and (C) cedar.

We then explored the MSPen(+) ability to characterize the two less closely related nongenuine (“fake”) but morphologically quite similar mahoganies, that is, andiroba and cedar. Figure 4 demonstrates that unique and very distinct chemical fingerprints were also obtained for cedar and andiroba, further highlighting the ability of MSPen(+) to provide effective wood typification. Andiroba (Figure 4B) exhibits a highly diverse array of ions within the vast *m/z* 200–1700 range. In contrast, the cedar profile (Figure 4C) is characterized by a much more diverse (and less abundant) poll of ions, predominantly within the *m/z* 466–566 range. Note that both “fake” mahoganies andiroba and cedar, although belonging to the same family, completely lack the very distinctive pair of ion packages (*m/z* 828–927 and *m/z* 1716–1799) of the “genuine” Brazilian mahogany (Figure 4A).

**FIGURE 5:**
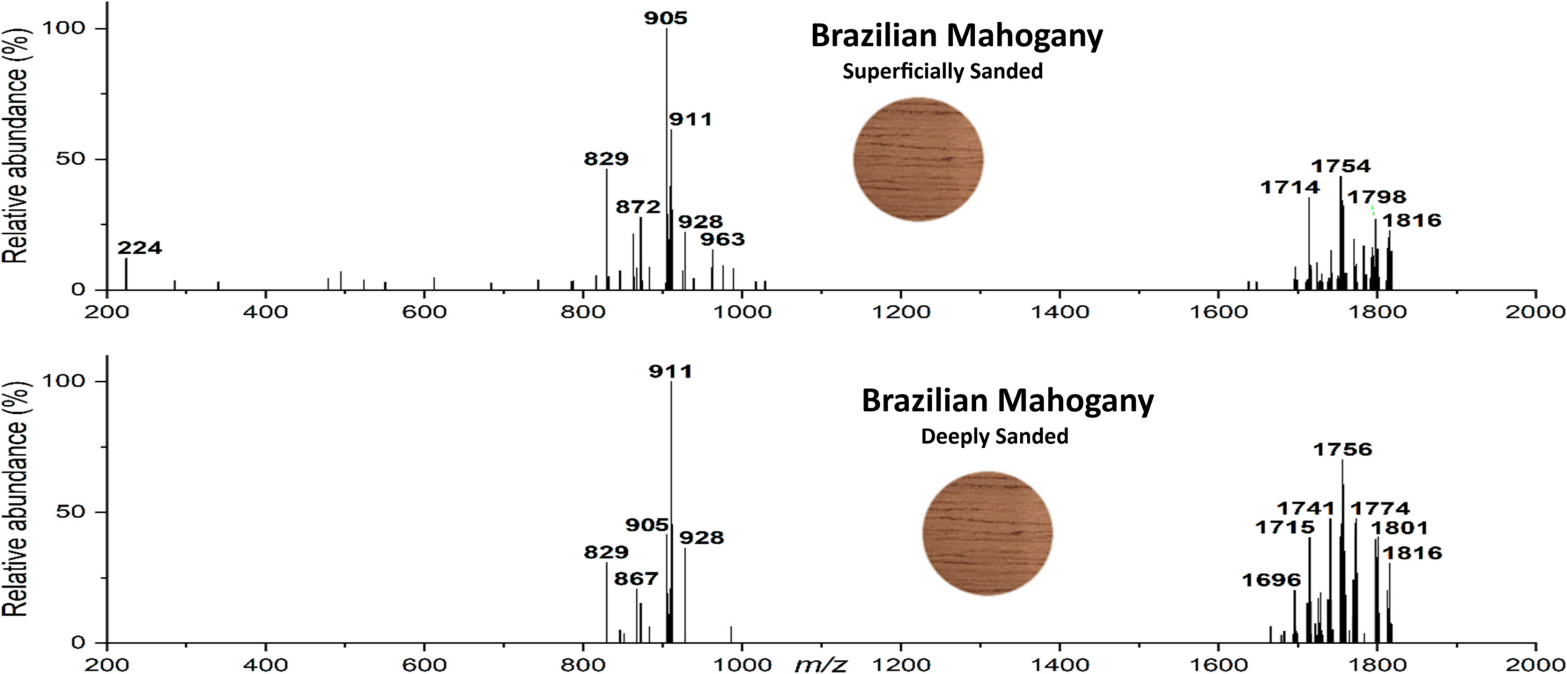
Representative MSPen(+) for (A) superficially‐sanded and (B) deeply sanded Brazilian mahogany.

It is widely known that the exposure of wood surfaces to the environment (sunlight and oxygen) triggers oxidation, which alters molecular composition and affect wood characteristics, including its hue [41]. To investigate the potential impact of surface oxidation on MSPen(+) profiles, a section of a Brazilian mahogany sample was either superficially or extensively sanded (to deeply remove its most oxidized utmost surface) and then analyzed (Figure 5). While a noticeable change in their abundances occurred, most of the highly characteristic MSPen(+) ions for the Brazilian mahogany remained detectable and predominant even after the exhaustive sanding process. This result indicates no crucial influence of wood oxidation or even thermal or hydrolysis degradation for MSPen(+) wood typification, and that only superficial sanding aimed to remove impurities or eventual varnish or waxing products should be recommended before analysis. Similarly, Cabral et al. [24] also reported little effect of aging on V_L_‐EASI‐MS data with similar chemical markers for a 20 year‐old Brazilian mahogany sample as compared with a 2 year‐old sample, showing that the wood makers persist when protected within the lignin–cellulose–hemicellulose structure.

### 3.2 Feature Selection and Multivariate Analysis

**FIGURE 6:**
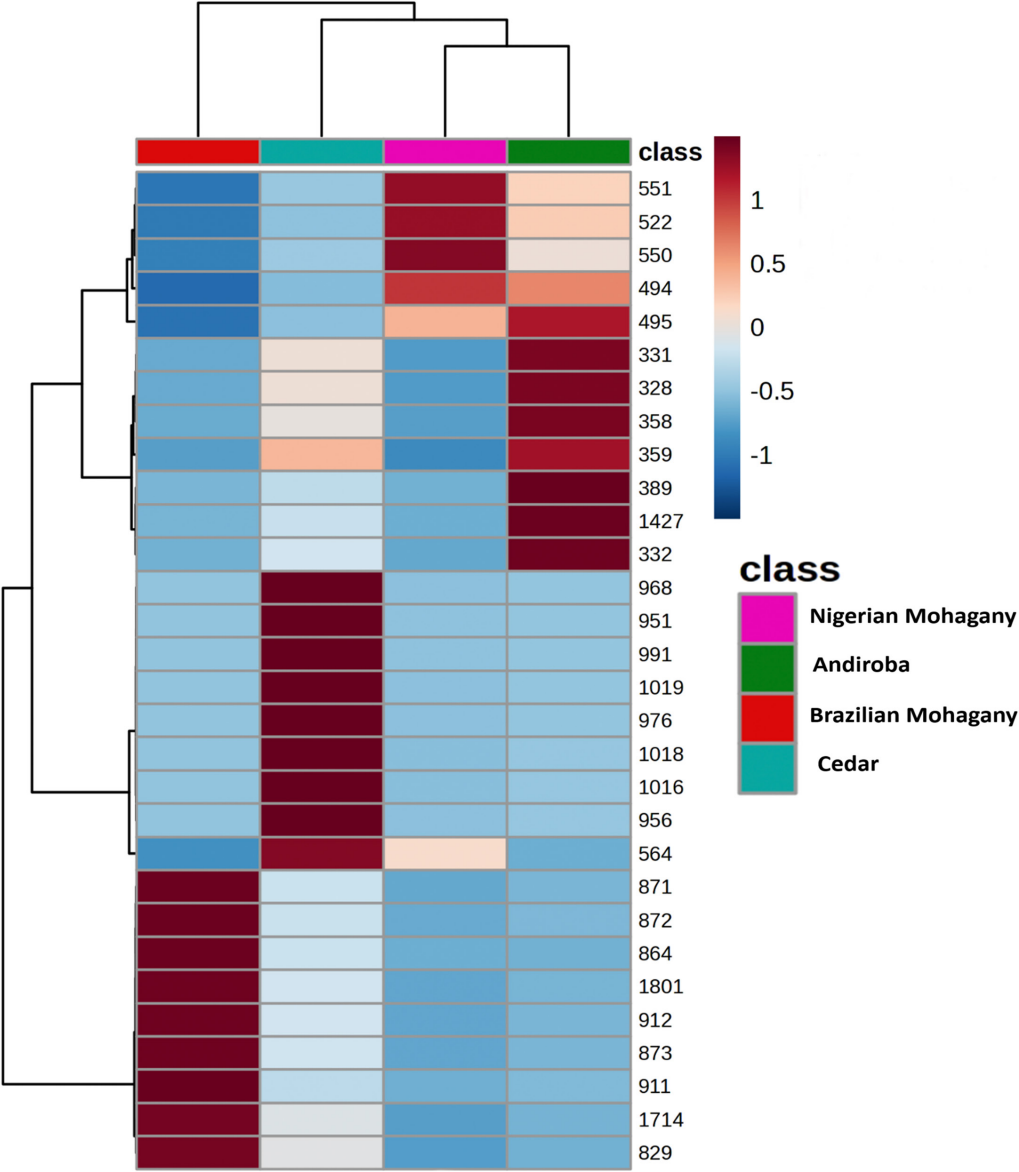
Hierarchical clustering heatmap displaying the top 30 discriminant features derived from Boruta analysis.

To statistically evaluate the performance of MSPen(+), feature selection and multivariate analysis were conducted for all samples except for the African Senegal mahogany due to the unavailability of biological replicates. A set of 59 variables for all wood data was selected by the Boruta algorithm, corresponding to 32.5% of the 181 initial features. Heatmap analysis was employed to visualize patterns of similarity among the wood samples, providing a heatmap (Figure 6) that reveals clear and intuitive representation of their distinct chemical profiles and relationships. It also provides visual discrimination using the top 30 features, where the dendrogram of the samples in the upper part of the heatmap shows the American Brazilian mahogany as the most distant for all four and African Nigerian mahogany and andiroba as the two more closely related.

**FIGURE 7:**
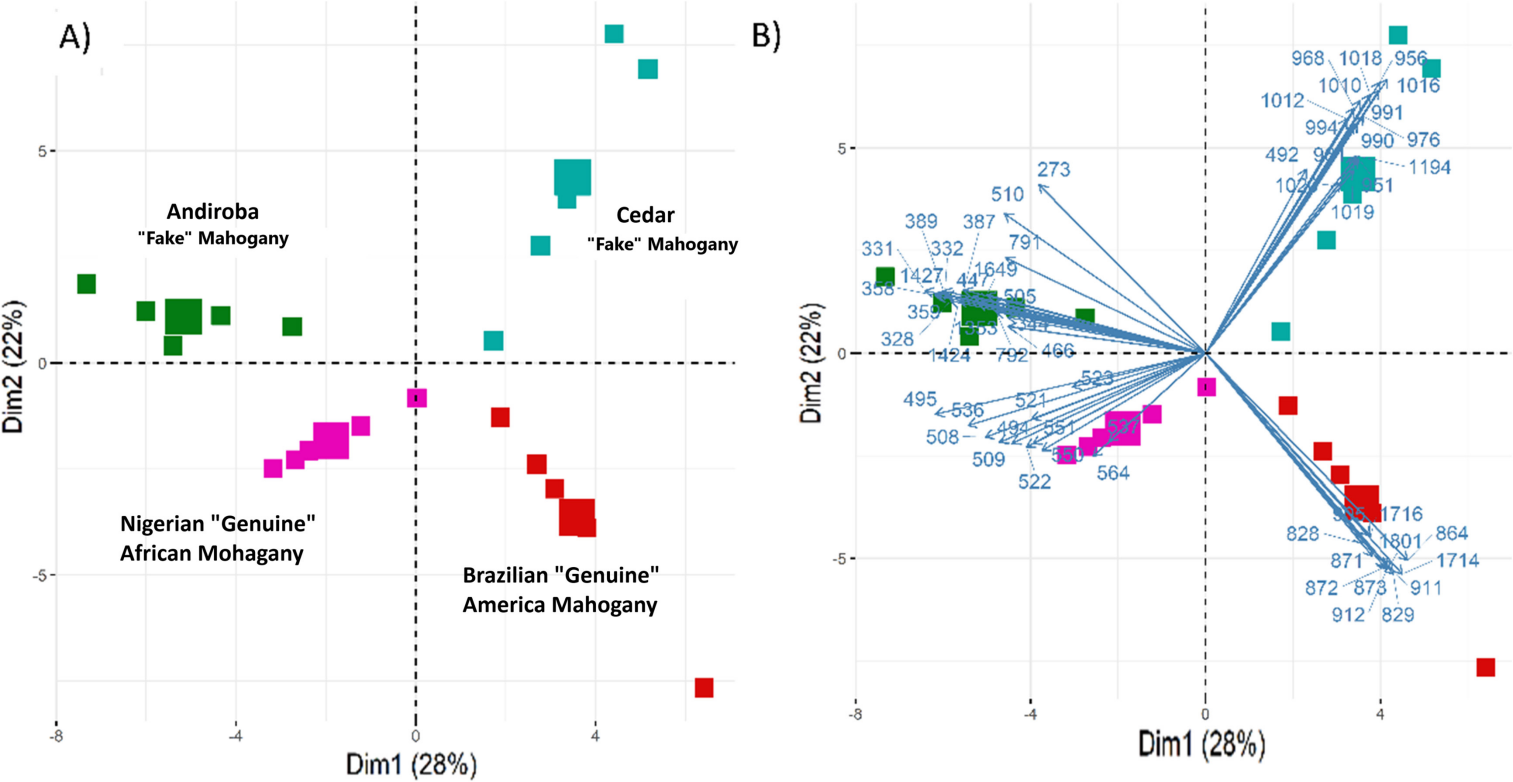
PCA performed on Boruta‐filtered MSPen(+) dataset displayed as (A) scores plot and (B) biplot.

PCA was also applied to assess the ability of MSPen(+) to differentiate the wood species. Figure 7A shows the 2D scores plot and 2D biplot (Figure 7B) of PCA performed on the Boruta‐filtered MS dataset, in which DIM1 and DIM2 explain 50% of the total variability. Overall, each class occupied a unique quadrant of the PCA, highlighting the great chemical diversity among these wood specimens and the robustness of the data acquired with the MSPen(+) to discriminate them. The DIM1 axis (28%) shows clear separation between Brazilian mahogany and cedar in the positive portion and Nigerian mahogany and andiroba in the negative portion. In Figure 7, separation and grouping of the classes can be understood via the distribution of the variables in the loadings represented by arrows. The closeness of the sample clusters in the quadrants suggests the least distance of Andiroba and African mahogany, whereas the Brazilian mahogany is the most divergent species, as also shown by the heatmap of Figure 6.

The integrated analysis of variable selection from the Boruta algorithm with hierarchical cluster and PCA biplot analysis also highlight a set of most diagnostic ions for each wood. As for Figure 3A or 4A, the main discriminatory ions for the Brazilian Mahogany were those of *m/z* of 828, 829, 864, 871, 872, 873, 905, 911, 912, 1714, 1716, and 1801. Also, as for Figure 3B, a contrasting set of ions of *m/z* of 494, 495, 508, 509, 522, 523, 536, 537, 550, 551, and 564 were identified as most discriminatory for the Nigerian mahogany. For andiroba, as noted in Figure 4B, the most discriminatory ions were those of *m/z* 328, 331, 332, 359, 358, 389, and 1427, and for cedar, as for Figure 4C, those of *m/z* 564, 951, 956, 968, 976, 991, 1016, 1018, and 1019.

Our investigation focused on the typification and is independent of metabolite identities, but note that a series of these fingerprinting ions have been previously attributed via high‐resolution MS and MS/MS as mexicanolide and phragmalin‐type limonoids [22, 24, 42]. They include those of *m/z* 828, 830, 872, and 846 for Brazilian and African mahoganies, *m/z* 864 for cedar, *m/z* 814, 788, 862, 820, 876, 804, 834, 818 for Nigerian mahogany, and *m/z* 760, 786, and 792 for andiroba.

## 4 Conclusion

As this exploratory study indicates, MSPen(+) provides characteristic and reproducible wood typification, even from closely related genera, in simpler, direct, fast, “touch&play,” nondestructive, and “spatially free” fashion. This technique should work therefore as an efficient tool to control illegal wood trading and forgery. An even more challenging test for MSPen(+) in wood typification, particularly for the mahogany family, would be, for instance, to check its ability to discriminate all five species of African mahoganies of the genus *Khaya* (Figure 1) as well all the three and most closely related American mahoganies from the genus *Swietenia* (Figure 1). Even more challenging would be to include samples from the seven species of Philippine mahoganies from the genera *Shorea* and *Parashorea*, the two species of Indian mahoganies ( *Toona ciliata* and *Chukrasia velutina* ), and the single species of Chinese mahogany ( *Toona sinensis* ). To collect the full set of these woods would be a tour‐the‐force endeavor, especially for certified samples in sufficient replicates, but if available, we predict, as judged by this present proof‐of‐principle study, that MSPen(+) will show outstanding discriminatory ability. We are currently perceiving this goal. But for now, and particularly for forensic science in Brazil, the MSPen(+) success to discriminate the endangered “genuine” Brazilian mahogany from two “fake” mahoganies (cedar and andiroba) as well as the other two “genuine” African mahoganies already demonstrated herein is of high relevance for forensic efforts to control illegal extraction and trading.

## Conflicts of Interest

The authors declare no conflicts of interest.

## Data Availability

The wood MS data used in this study can be found at https://doi.org/10.7910/DVN/MGILRM.
